# Long-Term Care in Fontan Circulation: Surveillance and Management of Fontan-Associated Liver Disease and Protein-Losing Enteropathy

**DOI:** 10.1007/s11886-026-02378-0

**Published:** 2026-05-19

**Authors:** Chaowapong Jarasvaraparn, Gary R. Schooler, Iván A. González, Salil Ginde, Alisha Mavis

**Affiliations:** 1 Division of Pediatric Gastroenterology, Hepatology and Nutrition, Indiana University, 705 Riley Hospital Drive, ROC 4210, Indianapolis, IN 46202 USA; 2 Department of Radiology, Cincinnati Children’s Hospital Medical Center, Cincinnati, OH USA; 3 Department of Pathology and Laboratory Medicine, Indiana University School of Medicine, Indiana, USA; 4 Department of Pediatric Cardiology, Medical College of Wisconsin, Milwaukee, WI USA; 5 Department of Pediatric Gastroenterology, Hepatology and Nutrition, Levine Children’s Hospital, Advocate Health, Charlotte, NC USA

**Keywords:** Fontan-associated liver disease, Fontan procedure, Protein losing enteropathy

## Abstract

**Purpose of Review:**

This manuscript reviews the clinical spectrum and management of Fontan-associated liver disease (FALD) and protein-losing enteropathy (PLE), examining how chronic venous hypertension leads to multisystem injury.

**Recent Findings:**

Liver fibrosis is now recognized as an early and nearly universal complication after the Fontan procedure, with cirrhosis affecting approximately 43% of patients by 30 years post-operation. Although post-2001 survival exceeds 90%, standard biomarkers and imaging frequently underestimate disease severity, as liver stiffness measurements are confounded by hepatic congestion. Liver biopsy remains the gold standard for staging.

**Summary:**

FALD is an inevitable consequence of Fontan physiology, characterized by sinusoidal congestion and progressive fibrosis, while PLE results from multifactorial gastrointestinal protein loss. The key clinical implication is the importance of a multidisciplinary approach, particularly when considering transition from isolated heart transplantation to combined heart–liver transplantation in advanced disease. Future research should prioritize standardized staging systems and targeted therapies to reduce lymphatic dysfunction and fibrosis progression.

## Introduction

The Fontan procedure, introduced in the 1970s by Fontan and Kreutzer [1, 2], diverts systemic venous return directly to the pulmonary arteries without a sub-pulmonary ventricle. Advances in technique and care have improved outcomes, with 10-year survival exceeding 90–95% for post-2001 operations [3]. The Fontan population continues to grow, estimated at 66 per million in 2020 and projected to reach ~ 79 per million by 2030 [4–6].

However, chronic venous hypertension and low cardiac output lead to multisystem injury, including Fontan-associated liver disease (FALD), a near-universal complication ranging from fibrosis to cirrhosis and hepatocellular carcinoma (HCC) [7]. The factors related to, and methods to abate FALD are lacking, due to a yet unclear understanding of FALD pathophysiology and disease progression.

### Fontan Physiology

The Fontan procedure is the final stage of surgical palliation for children with single-ventricle congenital heart disease. The procedure effectively reroutes systemic venous return directly to the pulmonary arteries creating a unique circulation of passive pulmonary flow without a sub-pulmonary ventricle. Fontan circulation is characterized by chronically elevated central venous pressure (CVP) and decreased cardiac output. Surgical evolution from atriopulmonary connection to lateral tunnel and extracardiac total cavopulmonary connection has reduced arrhythmias [8–10] and improved early survival. However, long-term complications remain common, including declining exercise intolerance, heart failure due to single ventricle dysfunction, protein-losing enteropathy (PLE), plastic bronchitis, arrhythmias, FALD, thromboembolism, kidney disease, and other forms of Fontan circulatory failure [3].

### Pathophysiology of FALD (Fig. 1)

**Fig. 1:**
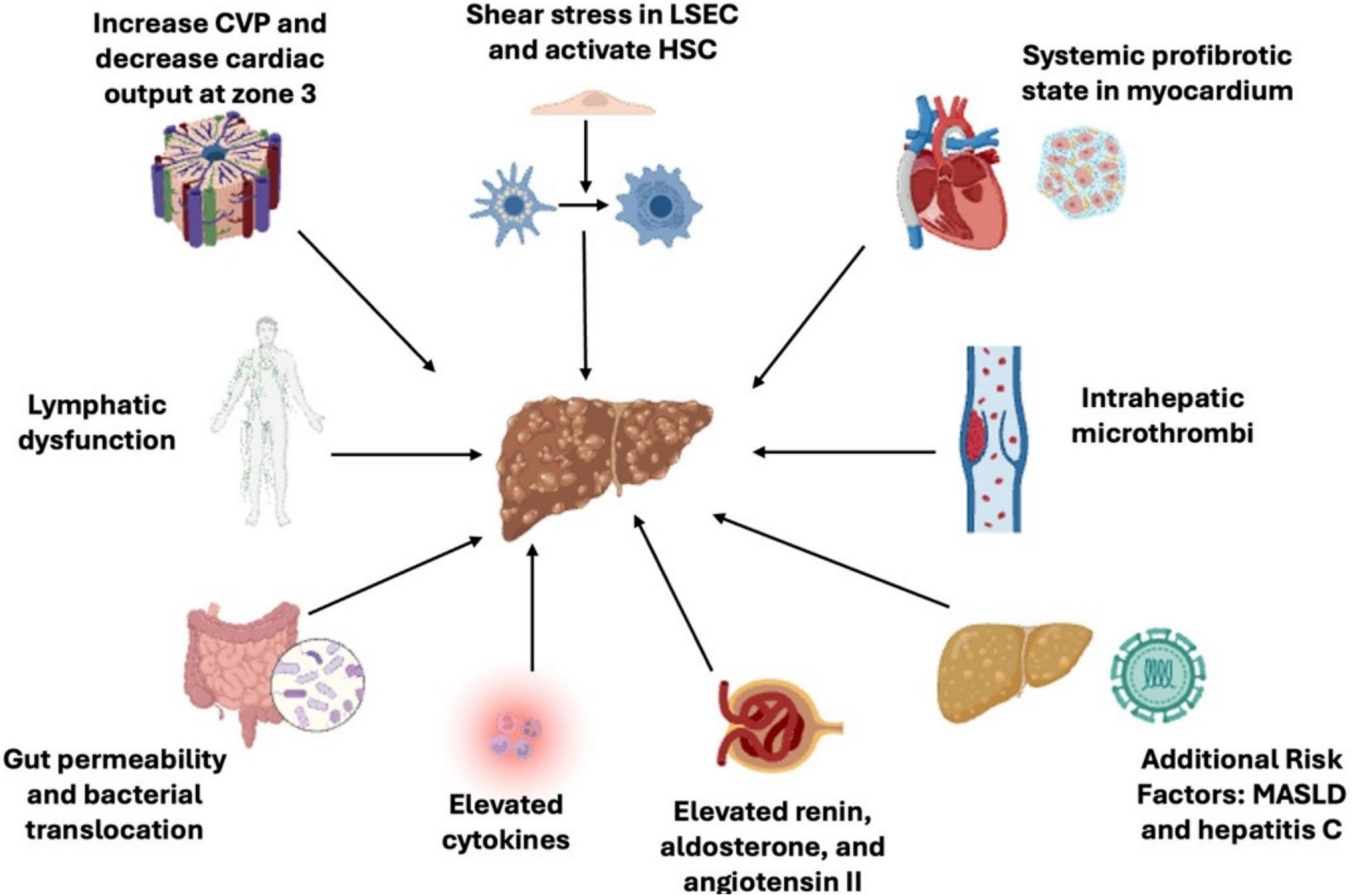
Pathophysiology of Fontan-associated liver disease. CVP; central venous pressure, LSEC; liver sinusoidal endothelial cells, HSC; hepatic stellate cells, MASLD; metabolic dysfunction-associated steatotic liver disease

1. Hemodynamic Changes and Circulatory Impact

This elevated CVP inherent within the Fontan circulation is directly transmitted to the hepatic veins and sinusoids, reducing the portal–hepatic venous gradient and impairing portal inflow [11]. Reduced cardiac output further compromises splanchnic perfusion, increasing reliance on hepatic arterial flow. Relative hypoxemia particularly affects zone 3 hepatocytes and promotes fibrogenesis [12]. Sinusoidal congestion and impaired hepatic venous outflow diminish the portal–hepatic gradient [13, 14]. Although increased hepatic arterial flow initially compensates (“hepatic arterial buffer response”), this mechanism fails once portal pressure exceeds ~ 20 mmHg, leading to ischemia and hepatocellular necrosis [11]. Notably, hepatic injury may begin before Fontan completion [15, 16]. Post-mortem studies reveal early fibrosis even within days/weeks after Fontan surgery, with sinusoidal fibrosis in 65–91% and portal fibrosis in 30–57% of patients [16, 17].

2. Cellular and Molecular Mechanisms

Sinusoidal congestion and mechanical stretch activate liver sinusoidal endothelial cells (LSECs) and hepatic stellate cells (HSCs). HSCs respond to mechanical stress by producing fibronectin and extracellular matrix components, initiating fibrosis [18, 19]. Strained LSECs release chemokines such as CXCL1 [20], promoting inflammation and platelet aggregation, which exacerbates portal hypertension and fibrogenesis [18, 21]. Importantly, fibrosis may develop with minimal inflammatory infiltrate, consistent with a predominantly mechanical/injury-driven process.

3. Systemic Profibrotic Environment

FALD reflects not only hepatic congestion but also a systemic profibrotic state. Myocardial and renal fibrosis have been observed in Fontan patients, and circulating fibrogenic mediators—including matrix metalloproteinases, tissue inhibitors, and growth differentiation factor-15—correlate with cardiac and hepatic fibrosis [12, 22].

4. Inflammation, Lymphatics, and the Microbiome

Although mechanical congestion predominates, chronic low-grade inflammation appears contributory. Elevated IL-6, TNF-α, and β2-microglobulin levels are reported in stable Fontan patients [23]. Recent single-cell RNA sequencing in adolescents with Fontan physiology identified increased endothelial cells and lymphocytes, particularly NK and T-cells, suggesting immune-mediated progression [24]. Lymphatic congestion leads to hepatic edema and fibrosis [25], with increased hepatic lymphangiogenesis similar to cirrhosis [26]. Elevated lymphatic pressure contributes to protein-losing enteropathy [27]. Gut hypoperfusion and permeability may drive systemic inflammation and bacterial translocation [25, 28]. The microbiome’s contribution remains an emerging research focus.

5. Neurohormonal and Thrombotic Contributions

Fontan patients demonstrate neurohormonal activation, including elevated renin–angiotensin–aldosterone levels, which promote collagen synthesis and fibrosis [12, 23]. A prothrombotic environment with intrahepatic microthrombi also contributes to fibrogenesis. In a murine model of congestive hepatopathy, partial inferior vena cava ligation induced sinusoidal thrombosis, HSC activation, hepatic fibrosis, and increased portal pressure [18]. Anticoagulation strategies are being investigated as potential therapies.

6. Additional Risk Factors

Comorbid factors—including prior hepatitis C exposure (particularly in recipients of blood products prior to 1992) [29], hepatotoxic medications such as amiodarone, and metabolic risk such as obesity—may accelerate FALD progression. Prevention focuses on optimizing metabolic health, minimizing hepatotoxic agents, and limiting alcohol use.

### Risk Factors Associated With Advanced FALD

Diagnosing FALD is challenging, leading to variable prevalence estimates. Reported cirrhosis rates differ by criteria, with one study showing 55% incidence among adult Fontan survivors at ~ 18.4 years post-Fontan [30], while another reported 10-, 20-, and 30-year freedom from cirrhosis of 99%, 94%, and 57% [31]. Liver fibrosis appears nearly universal by 10 years [32], correlating with Fontan duration [17, 31–35] and pre-Fontan hemodynamics [15, 16]. Fibrosis may precede Fontan completion [33, 36, 37]. Patients with advanced fibrosis had higher Fontan pressures (16 vs 13 mmHg, p = 0.01) [38] and lower platelet count [39, 40]. Heart failure, arrhythmias [41], atrioventricular valve regurgitation, advanced age, and elevated central venous pressure are associated with cirrhosis and hepatocellular carcinoma [42, 43].

### Clinical Presentation of FALD

FALD is often subclinical for years, making early surveillance essential. Symptoms vary by stage and whether hepatic congestion or low cardiac output predominates [44]. Chronic congestion may cause hepatomegaly, ascites, hypoalbuminemia, and indirect hyperbilirubinemia, whereas chronic ischemia leads to cholestatic enzyme elevations [45]. Laboratory tests may remain normal until advanced disease. Portal hypertension occurs in both congestive and fibrotic stages and the VAST score (Varices, Ascites, Splenomegaly, Thrombocytopenia) is used to predict adverse events [46]. Varices occur in up to 40% of adults [47, 48]. Brain natriuretic peptide (BNP) helps distinguish cardiac vs hepatic ascites (> 364 pg/mL sensitivity 98%, specificity 99%) [49].

### Diagnosis of FALD

#### Histologic Evaluation of FALD

**Fig. 2:**
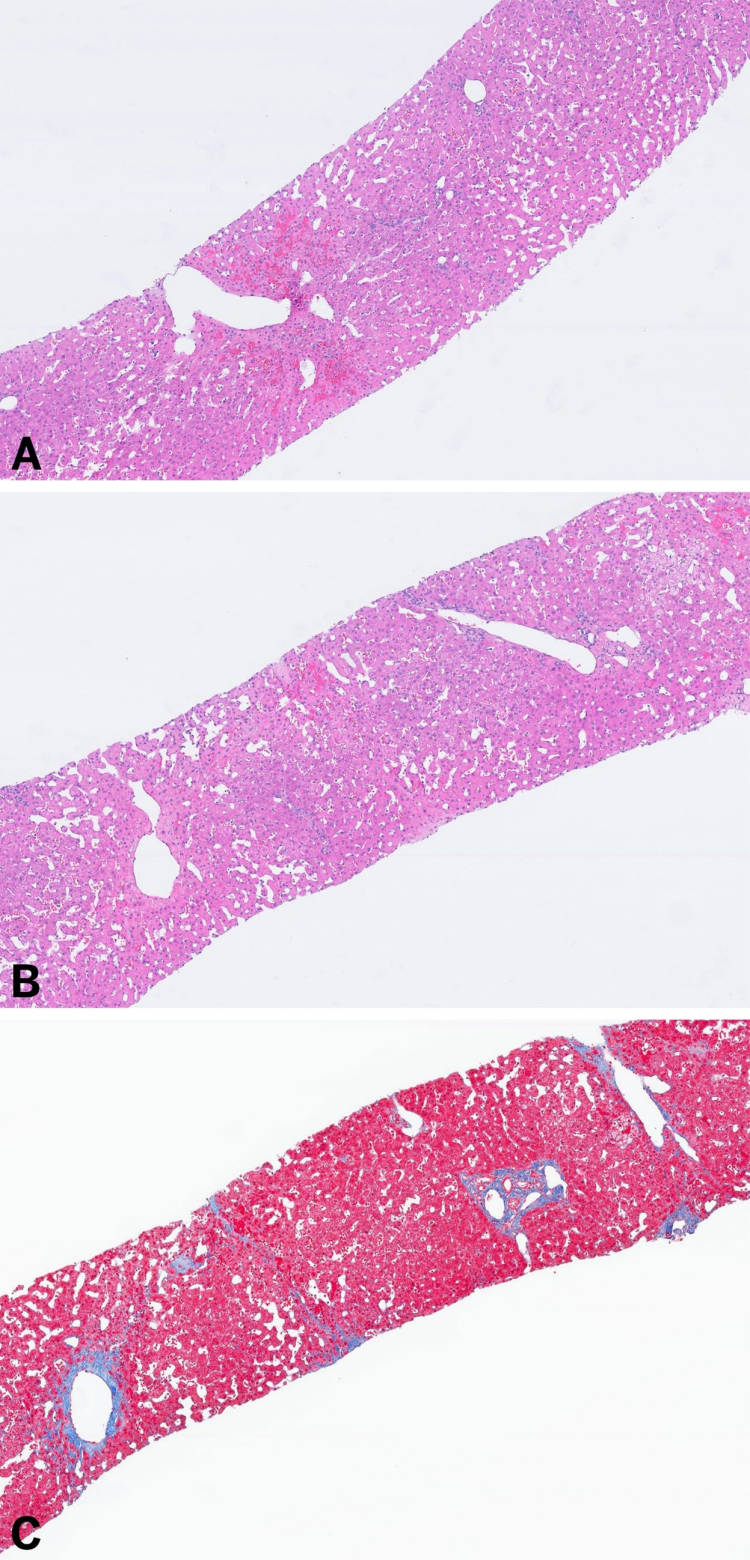
Liver biopsy showing the classic findings of congestive hepatopathy including a sinusoidal dilatation with hepatic plate thinning and focal central zone congestion (**A and B,** H&E). Trichrome Masson stained slide shows in this biopsy only perisinusoidal and pericentral fibrosis (**C**)

The cardinal histologic feature of FALD is congestive hepatopathy which is characterized by a sinusoidal dilatation and congestion beginning in zone 3 (Fig. 2), with central and perisinusoidal fibrosis, and up to 90% of cases demonstrating portal fibrous expansion [32, 50, 51].

**Fig. 3:**
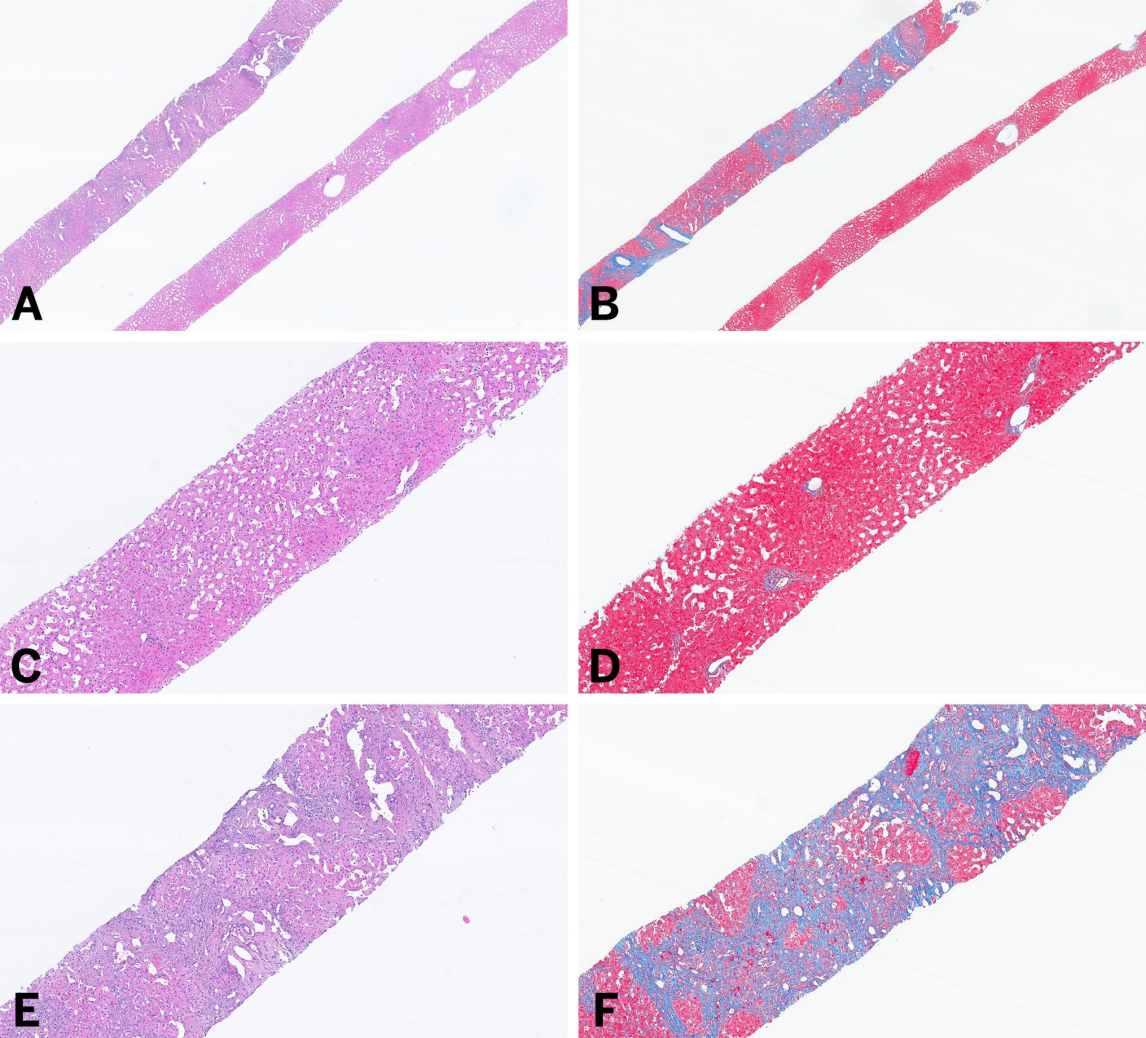
Two cores of liver showing an heterogenous pattern of fibrosis, with the upper core showing evidence of advanced fibrosis while the other show no increased in fibrosis (**A,** H&E, and **B,** Trichrome Masson). **C** (H&E) and **D** (Trichrome Masson), shows representative photograph of the bottom core showing classic features of congestive hepatopathy without increased in fibrosis; and **E** (H&E) and **F** (Trichrome Masson) shows a cirrhotic architecture with prominent sinusoidal dilatation and dilated vessels

Because fibrosis in FALD is heterogeneous and predominantly central, traditional scoring systems are inadequate (Fig. 3). The congestive hepatic fibrosis score (CHFS) (Table 1) correlates with elevated right atrial pressure and chamber dilation [52, 53], and it is increasingly used for post-Fontan fibrosis assessment [54, 55]. A modified Ishak-CHFS system has also been proposed, though neither scoring approach has been prospectively validated for predicting outcomes such as transplant-free survival [54, 56–58].

**Table 1:** Fibrosis scoring systems for Fontan associated liver disease

Score	Congestive hepatic fibrosis score (CHFS) [52]	Modified Ishak CHS (ICHF) [57]
0	No fibrosis	
1	Central zone fibrosis	
2A	Central zone and periportal fibrosis, with fibrosis accentuation in the central zone	
2B	Central zone and periportal fibrosis, with fibrosis accentuation in the portal zone	
3	Bridging fibrosis	Fibrous expansion of most portal areas with occasional portal to portal bridging
4	Cirrhosis	Fibrous expansion of portal areas with marked bridging (portal to portal and portal to central)
5		Marked bridging fibrosis with occasional nodules (incomplete cirrhosis)
6		Cirrhosis

Liver biopsy remains the gold standard for fibrosis assessment and is often used for surveillance or transplant evaluation. However, its routine use, particularly in children, is limited by bleeding risk related to anticoagulation, congestion, and thrombocytopenia. Transjugular biopsy (TJLB) is preferred due to lower bleeding risk and the ability to obtain hemodynamic data [55]. In one series, percutaneous biopsy had a 7.4% bleeding rate in Fontan patients [59] and fibrosis was underestimated in 40% of pre-transplant biopsies compared with explants [60]. European Association for the Study of the Liver (EASL) guidelines recommend TJLB with ≥ 2 passes in Fontan patients [61].

### Imaging in FALD

FALD produces characteristic but heterogeneous morphologic changes that are increasingly recognized on imaging. A major challenge is the poor correlation between radiologic abnormalities and histologic fibrosis [54, 62]. Hepatic congestion alone can generate a nodular contour mimicking cirrhosis, leading to potential overestimation of severity when relying solely on liver morphology [63].

### Ultrasound (US) Imaging in FALD

US is a widely available first-line modality but has limited sensitivity for early fibrosis. US typically demonstrates hepatomegaly, dilated inferior vena cava (IVC) and hepatic veins (HVs), and heterogeneous parenchyma reflecting congestion and fibrosis [64]. Doppler findings include dampened or absent triphasic HV waveforms and abnormal portal venous velocities, consistent with elevated central venous pressure or portal hypertension [64]. A retrospective cohort of 131 Fontan patients identified heterogeneous echotexture, lobar redistribution (posterior right lobe atrophy with left lateral and caudate hypertrophy), and surface nodularity as common findings [65]. These features did not predict mortality [65], although surface nodularity has been associated with Fontan duration and fibrosis severity in some cohorts [66]. Portal venous flow velocity may serve as a noninvasive marker of portal hypertension in children [67].

### Computed Tomography (CT) and Magnetic Resonance (MRI) in FALD

CT and MRI provide better characterization of parenchymal changes, vascular congestion, and portal hypertension. Both modalities commonly show hepatomegaly, IVC and HV dilation, lobar redistribution, heterogeneous parenchyma, and surface nodularity, with more frequent detection of varices and umbilical vein recanalization versus US [65]. On CT, heterogenous parenchymal attenuation and enhancement along with reflux of contrast into HVs/IVC may be seen [68]. Both CT and MRI may show heterogeneous enhancement during the portal venous phase due to slow portal vein circulation, which tends to become more homogeneous on delayed imaging [3–5 min post-contrast] [68].

**Fig. 4:**
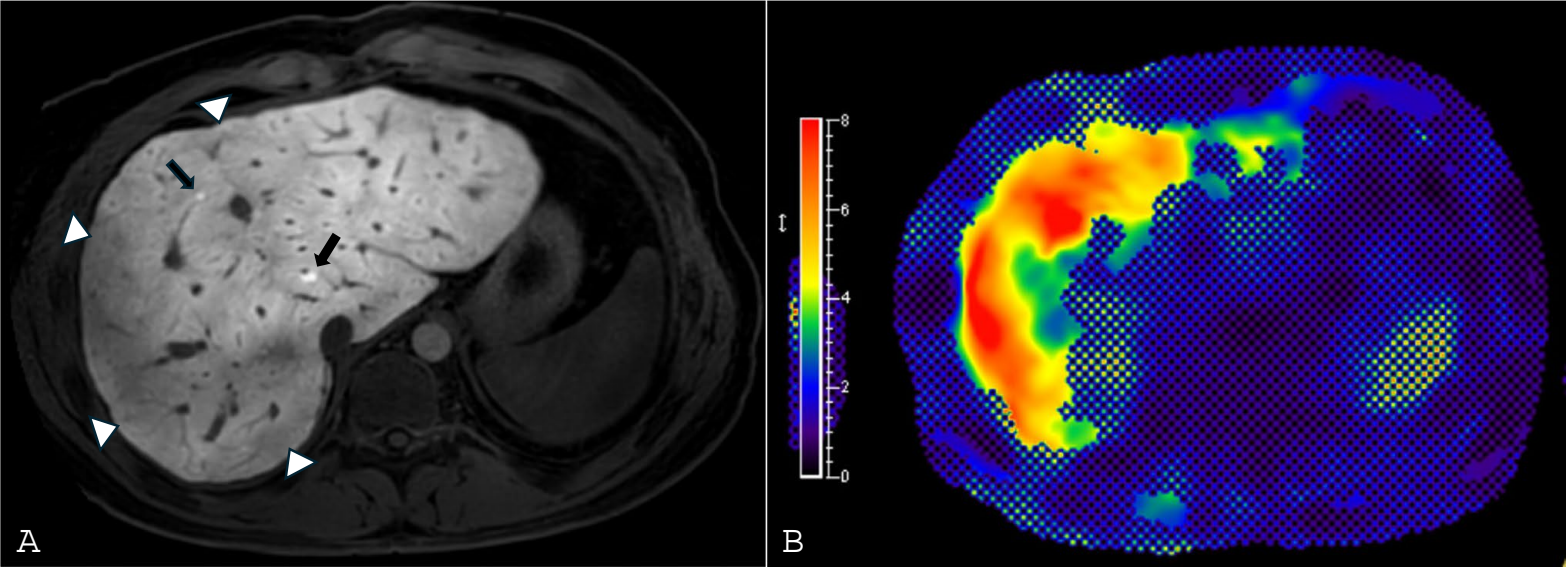
21-year-old female status post Fontan palliation with Fontan associated liver disease. **A**) Axial hepatobiliary phase MR image, obtained after administration of gadoxetate disodium, demonstrates a peripheral nodular contour of the liver with some areas of relative hypoenhancement of the liver parenchyma, predominantly in the liver periphery (arrowheads) representing areas of fibrosis. The left lobe of the liver is also mildly hypertrophied. Two small lesions that are retaining the hepatobiliary contrast agent are present and consistent with FNH-like lesions (*arrows*). **B**) Stiffness map from MR elastography reveals abnormally elevated stiffness of the liver parenchyma (indicated as higher stiffness parenchyma colored red per the scale on the left side of the image). This patient’s mean liver stiffness was 3.5 kPa, which is abnormally elevated

MRI offers superior soft-tissue contrast, demonstrating T2 and diffusion-weighted hyperintensity and characteristic zonal or reticular enhancement [64, 69, 70] (Fig. 4A), which correlates with elevated HV pressures, advanced fibrosis, and longer Fontan duration [37]. Left-lobe perfusion defects may signify more severe portal hypertension in children [71]. Hepatobiliary contrast agents (e.g., gadoxetate disodium) improve functional and fibrotic assessment [64].

CT provides rapid assessment but involves radiation, particularly relevant in young congenital heart disease patients [72]. MRI is preferred when feasible but may be limited by implantable cardiac device (i.e. permanent pacemakers) compatibility and cost. Importantly, qualitative CT/MRI findings typically manifest only with moderate-to-advanced fibrosis [64]; thus, imaging should be integrated with quantitative stiffness measures and clinical evaluation.

### Monitoring in FALD

#### Laboratory-Based Monitoring of FALD

Laboratory abnormalities are common in FALD but correlate poorly with fibrosis severity [51]. In one cohort, ALT and bilirubin were elevated in 15%, INR in 50%, thrombocytopenia in ~ 25%, and GGT in all patients [54]. Noninvasive fibrosis scores used in other liver diseases—including AST platelet ratio index (APRI), Fibrosis-4 (FIB-4), Forns fibrosis index, and Model for End Stage Liver Disease (MELD)—lack validation in the Fontan population (Table 2). APRI and FIB-4 show limited diagnostic accuracy, though both associate with mortality in adults with Fontan physiology [73]. A Japanese study reported higher FALD incidence in patients with FIB-4 > 0.17 at 2 years post-Fontan (45.6% vs. 3.9%, p = 0.002) [74]. A meta-analysis showed differences in platelet count, APRI, and FIB-4 between mild and severe fibrosis [75]. Forns fibrosis index shows better performance (AUROC ≈0.79) [34].

**Table 2:** Summary of noninvasive laboratory-based scoring systems that can be used to estimate fibrosis stage

Non-invasive scores	Formula	Cut point to detect stage 3–4 or cirrhosis on imaging	AUC	Sensitivity	Specificity	Ref
APRI	(AST in IU/L)/(AST Upper Limit of Normal in IU/L)/(Platelets in 10^9^/L)	≥ 0.6 ≥ 1.5 > 0.35	0.69 0.56 0.7	27.3% 65.8%	93.5% 65.4%	[39] [34] [38]
FIB-4	(Age* x AST)/(Platelets x √(ALT))	≥ 0.74 > 0.57	0.69 0.67	24.2% 71.8%	90.3% 45.2%	[39] [38]
Forns index	7.811- 3.131 × ln(platelet count) + 0.781 × ln(GGT) + 3.467 × ln(age)—0.014(cholesterol)	< 4.21	0.79			[34]
AST/ALT ratio	AST/ALT ratio	≥ 1	0.57			[34]
Pohl score	Positive; AST/ALT ratio ≥ 1 in combination with a platelet count of < 150,000	Positive	0.70			[34]
MELD score	0.957 × ln(Cr) + 0.378 × ln(bilirubin) + 1.120 × ln(INR) + 0.643	> 8.5	0.61 (p = 0.07)	63.2%	46.2%	[38]
MEDL-XI score	5.11 Ln(bilirubin) + 11.76 Ln(creatinine) + 9.44	Correlation coefficient = 0.4 (p = 0.003)	Failed to predict fibrosis			[76]

MELD-XI, unaffected by anticoagulation, correlates with fibrosis [76] and clinical short-term survival in cirrhotic adults on anticoagulant therapy [77]. MELD-XI also predicts post heart transplant outcomes in patients with Fontan [78–80]. Other serum markers (e.g., hyaluronic acid, type IV collagen, M2BPGi) remain unreliable in FALD [81].

### Imaging-Based Monitoring of FALD

#### Elastography in FALD

**Fig. 5:**
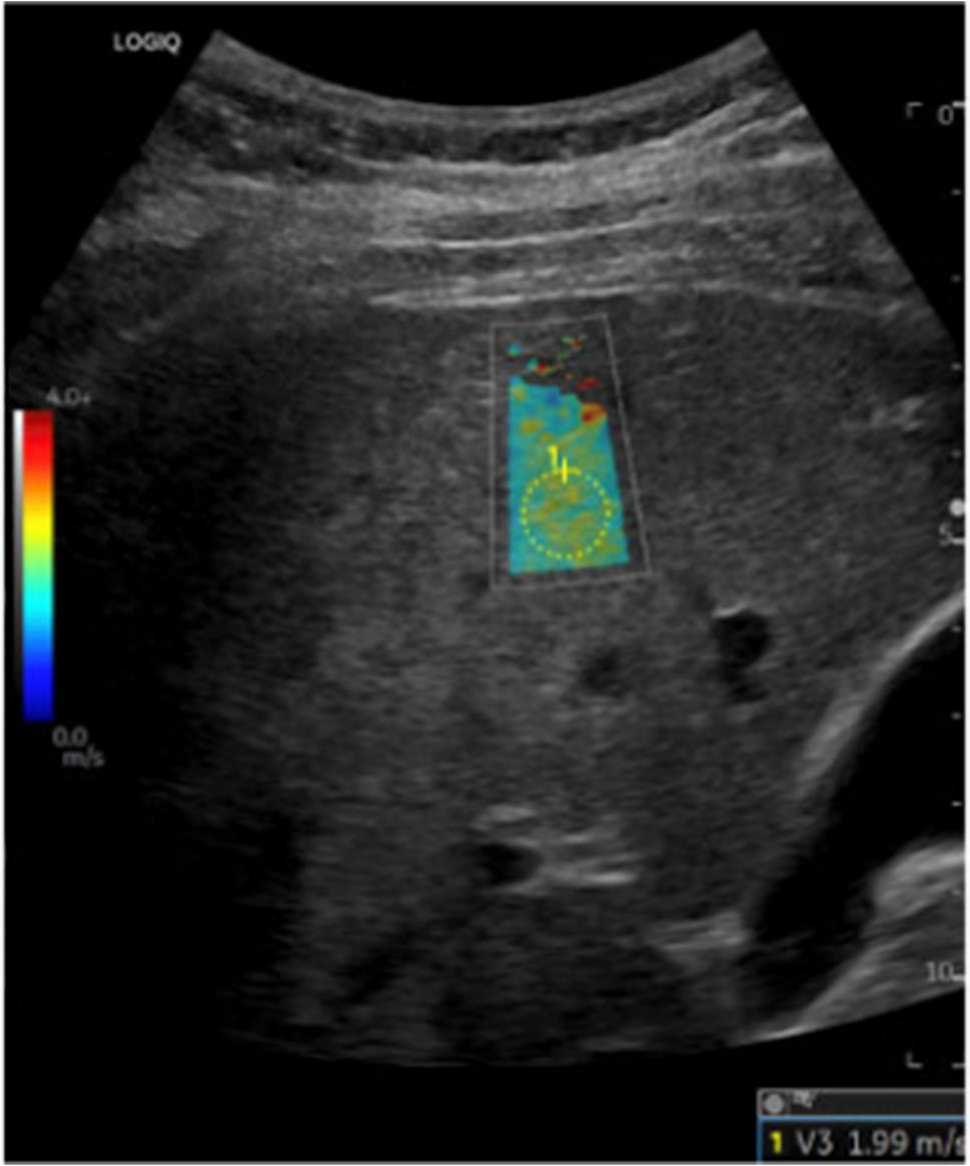
Transverse ultrasound elastography image of the right lobe of the liver in a 20-year-old male patient who is status post Fontan palliation. The image shows mild coarsening of the hepatic parenchymal architecture with an elastography shear wave speed of 1.99 m/s. The median shear wave velocity for this patient was 1.9 m/s, which is elevated

Elastography—shear-wave elastography (SWE) and magnetic resonance elastography (MRE)—noninvasively measures liver stiffness, but stiffness in Fontan patients reflects both fibrosis and venous congestion. Nearly all post-Fontan patients demonstrate elevated stiffness due to chronic congestion, complicating fibrosis assessment [64] (Fig. 4B, Fig. 5). MRE correlates with biopsy scores and Fontan pressures; values > 4–4.5 kPa and Fontan pressure ≥ 14 mmHg suggests advanced disease or failing circulation [57, 82]. SWE results are variable, with modest discrimination of severe disease in some studies [83], and no difference in others [75].

Multiple studies have shown that liver stiffness correlates inversely with cardiac index and ejection fraction and positively with Fontan pressure, CVP, and time since surgery [82, 84]. While MRE and SWE are less reliable in staging fibrosis in FALD [85, 86], MRE still correlates with outcomes such as portal hypertension and Fontan failure [82, 87, 88]. Progression of liver stiffness measurement (LSM) via MRE has shown associations with adverse clinical outcomes including death, listing for heart–liver transplant, engagement of palliative care, hospitalization, and need for paracentesis [89]. Longitudinal increases (> 0.3 kPa/year) predict mortality [82], and may assist in deciding when to pursue invasive testing (e.g., biopsy or catheterization) [61].

#### FibroScan in FALD

Transient elastography (TE) from FibroScan often overestimates fibrosis severity in FALD due to congestion. Fibroscan helps in early detection of FALD, distinguishing more advanced disease (e.g. using cutoff values ~ 20–25 kPa), predicting the risk of complications (ascites, varices, hepatocellular carcinoma, transplant need), and monitoring over time when combined with clinical/laboratory parameters [90]. The real benefit of FibroScan over other modalities is accessibility in the clinic space.

TE values are typically high (~ 28 kPa) in pediatric and adult Fontan patients [33]. Another adult study showed combining TE from FibroScan with platelet count, such as the FonLiver risk score (AUC 0.81), identifies severe fibrosis [91]. FonLiver risk score = 0.104647 × LSM (kPa) − 0.0077689 × Platelet count (10^3^/mL) − 1.340572.

Longitudinal assessment of LSM during follow-up could help monitor patients and predict clinical outcomes. Thus, in surveillance for FALD, LSM from several modalities can be considered and easily performed when available as an adjunct to a liver imaging [61].

APRI, AST to Platelet Ratio Index; FIB-4, Fibrosis-4; MELD, Model for End-Stage Liver Disease; MELD-XI, Model for End-Stage Liver Disease excluding International Normalized Ratio.

#### Liver Nodules in FALD

Liver nodules are common in FALD, ranging from benign regenerative and hyperplastic lesions to adenomas and malignancies, including hepatocellular carcinoma (HCC) and cholangiocarcinoma [92]. Liver masses occur in 20–48% of patients [69, 93–95] and HCC develops in ~ 0.18–1.3%, on average about 22 years post-Fontan, including in non-cirrhotic patients [96, 97]. MRI is preferred for lesion detection and characterization, with CT reserved for patients unable to undergo MRI. Ultrasound has limited sensitivity for nodule detection in this population [69].

Focal nodular hyperplasia (FNH)-like lesions are the most frequent benign nodules, especially in children, and are likely related to compensatory arterialization from venous outflow obstruction [98, 99]. On MRI, they are iso- to mildly hyperintense on T2-weighted images, iso- to mildly hypointense on T1-weighted images, hypervascular in the arterial phase, and isoenhancing in the portal phase; with hepatobiliary agents, they typically retain contrast [100] (Fig. 4A).

**Fig. 6:**
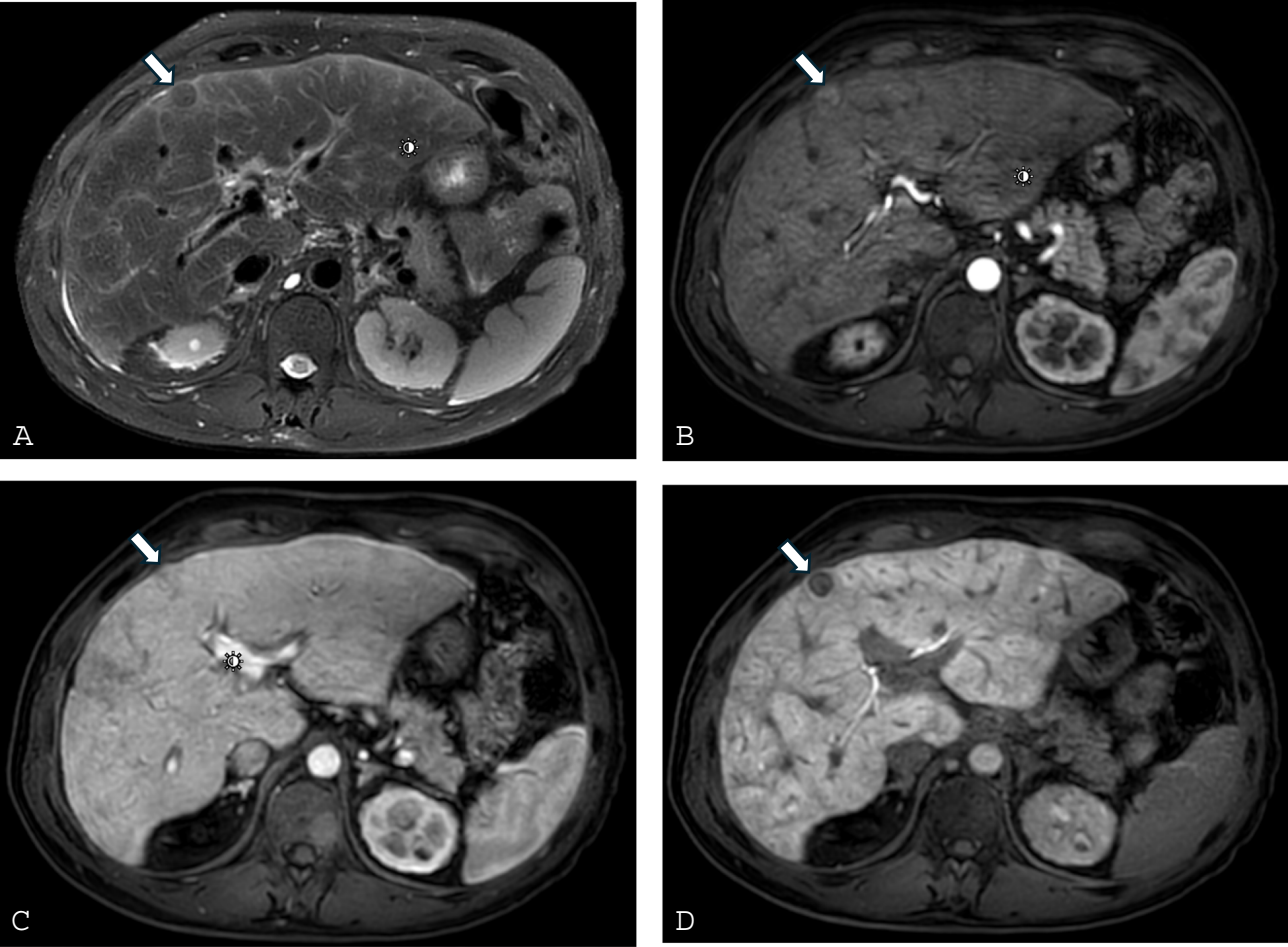
42-year-old male status post Fontan palliation with Fontan associated liver disease. Axial fat-suppressed T2-weighted (**A**) MR image shows peripheral T2-hyperintense reticulation typical of FALD representing sequalae of hepatic congestion and fibrosis. A lesion is present in the anterior right hepatic lobe (*arrow*) that is T2-hypointense. Dynamic post-contrast fat-suppressed T1-weighted MR imaging during the arterial phase (**B**) shows arterial phase hyperenhancement with subsequent washout on the portal venous phase (**C**). The lesion does not retain the hepatobiliary contrast agent on the hepatobiliary phase (**D**) but does have a thin rim of peripheral retention. The lesion was biopsied and proven to be HCC

Differentiating benign from malignant nodules is challenging due to overlapping imaging features [101]. Liver Imaging Reporting and Data System (LI-RADS) should not be applied in Fontan patients [102], as lesions show mixed morphology and benign lesions can display enhancement characteristics traditionally associated with malignancy in LI-RADS [98]. While overlap in benign and malignant lesion imaging features limit their use in isolation, some imaging features may support additional lesion scrutinization, including disproportional/rapid lesion growth and lack of hepatobiliary contrast agent retention on the hepatobiliary (20 min) phase of imaging on MRI (Fig. 6). Alpha-fetoprotein (AFP) levels can aid diagnosis, although they may be normal in 20–25% of HCC cases in Fontan patients [97, 103]. Elevated AFP (> 400 ng/mL) favors malignancy [98]. Ambiguous lesions warrant short-term imaging follow-up in 3–6 months and/or biopsy. Multidisciplinary review is critical.

### Portal Hypertension

Portal hypertension (PHTN) is an important feature of FALD and contributes to adverse outcomes, including ascites, variceal hemorrhage, and encephalopathy [46]. Hepatic venous pressure gradient (HVPG) measurement is limited in Fontan physiology due to venovenous collaterals [11]. Both wedged and free hepatic venous pressures are typically elevated, whereas HVPG remains ≤ 5 mmHg unless parenchymal disease is present; HVPG > 5 mmHg suggests intrinsic hepatic pathology [11]. HVPG does not predict fibrosis severity or prognosis in Fontan patients [61]. Severe PHTN appears uncommon, though data remain limited.

Ascites may be hepatic or cardiac in origin; serum albumin–ascites gradient, ascites protein, and brain natriuretic peptide (BNP) assist in distinguishing etiologies. BNP > 364 pg/mL supports cardiac ascites (sensitivity 98%, specificity 99%), whereas BNP < 182 pg/mL argues against it [49]. Ascites occurs in 4–58% of patients and is managed with sodium restriction, diuretics, and paracentesis [19].

Gastroesophageal varices (GEVs) occur in 9–38% of post-Fontan patients [37, 47, 48], usually 10–17 years post-surgery; bleeding is uncommon (5–6%) [30]. Upper-esophageal “downhill” varices result from elevated central venous pressure, whereas lower-esophageal varices reflect PHTN [61]. Low platelets (< 119 × 10⁹/L) predict GEVs in children (AUC 0.946) [104]. Varices correlate with worse outcomes [51], and EASL recommend screening endoscopy in patients with cirrhosis or PHTN in adults with Fontan procedure [61].

### Outcomes in FALD

Severity of FALD independently predicts worse long-term survival post-Fontan. In 512 patients, > 50% developed severe FALD by 35 years, tripling mortality (HR ~ 3.38) [41]. Meta-analysis of 2,466 patients showed OR ≈3.09 for death in patients with FALD [105]. Advanced fibrosis or cirrhosis with PHTN further worsens outcomes [39, 40], highlighting the need for early detection and surveillance. A recent multicenter study on HCC in Fontan patients (N = 58) reported a one-year survival rate of 79%. However, the study's conclusion stresses the critical role of screening for early-stage disease, which was associated with the highest survival [106].

### Surveillance of FALD

Fontan patients require lifelong FALD surveillance, though no universal guidelines exist. Expert consensus recommends individualized monitoring to enable early detection before HCC [44, 107]. Baseline evaluation includes biochemical tests, with repeat assessments every 2–3 years, increasing in frequency after 10 years post-Fontan. Imaging with US or MR elastography monitors fibrosis, while cross-sectional imaging detects focal lesions.

Liver biopsy is still the gold standard diagnosis of FALD [107]. The liver biopsy’s role as a routine surveillance tool 10–15 years after Fontan is debated [51] in Table 3. The procedure offers unmatched diagnostic detail but carries procedural risk, particularly in patients with elevated venous pressure. Current consensus favors a selective rather than routine approach, reserving biopsy for cases with diagnostic uncertainty, discordant non-invasive findings, or when results would directly influence management (e.g., transplant evaluation).

**Table 3:** Advantages and Limitations of Surveillance Liver Biopsy in FALD (10–15 Years Post-Fontan). **Key:** FALD, Fontan-associated liver disease; CHLT, Combined heart and liver transplant; IHT, Isolated heart transplant

Aspect	Advantages (Pros)	Limitations (Cons)
Diagnostic accuracy	- Gold standard for assessing fibrosis, and architectural distortion - Enables assessment of additional hepatic pathology (e.g., steatohepatitis, chronic hepatitis, iron overload, viral hepatitis)	- Sampling error due to heterogeneous fibrosis pattern in FALD - Interobserver variability in grading congestion and fibrosis - May under/overestimate disease severity if biopsy not targeted to representative regions
Clinical decision-making	- Helps stage FALD and guide timing of surveillance imaging, transplant referral, or need for CHLT vs IHT	- Limited evidence that biopsy findings change management in stable patients
Monitoring progression	- Can document fibrosis progression compared to prior biopsies - Useful for correlating histologic severity with non-invasive tests (elastography, serum biomarkers)	- Invasive nature makes repeated biopsies impractical for longitudinal monitoring - Fibrosis may progress slowly, reducing utility of serial sampling
Safety considerations	- Generally, well tolerated when performed percutaneously or transjugular by experienced teams	- Risk of bleeding, especially in patients with elevated central venous pressure, thrombocytopenia, or coagulopathy - Requires anesthesia/sedation in many pediatric or young adult patients - Potential for post-procedure pain or transient hemodynamic instability

### Transplantation Strategies and Outcomes in FALD

No established medical therapies exist for FALD. As Fontan survival improves, more patients are developing FALD, thus increasing transplant listing [108, 109]. Management centers on deciding between isolated heart transplantation (IHT) or combined heart–liver transplantation (CHLT), as liver transplantation alone is generally contraindicated due to elevated CVP and limited cardiac reserve [51]. CHLT is indicated in advanced hepatic dysfunction, portal hypertension (PHTN), or hepatocellular carcinoma (HCC) [50].

Retrospective studies demonstrate IHT is feasible in children and young adults with mild or compensated FALD. Post-IHT imaging often shows stabilization or regression of hepatic abnormalities, with 1- and 5-year survival comparable to other indications [110, 111]. Another pediatric study revealed no significant difference in 1-year all-cause mortality after IHT between patients with and without cirrhosis [35]. Nevertheless, continued liver surveillance is essential, as FALD features may persist, and risk for HCC remains [112, 113].

CHLT outcomes are increasingly favorable, with 1-year survival > 85–90% and 10-year survival ~ 80% [114–116]. Another pediatric study showed posttransplant mortality was similar after CHLT and IHT (18% vs. 11%; p = 0.64) at a median follow-up of 17 months and found imaging evidence of cirrhosis was associated with worse outcomes after IHT [117]. Compared with IHT, CHLT demonstrates lower rejection rates and improved graft survival, reflecting immunologic benefits such as donor chimerism, alloantibody clearance, and T-cell modulation [114, 115, 118–121].

Registry data show 5-year survival is higher after CHLT (≈86%) than IHT (≈52%) in patients with advanced FALD [122–124]. The FALD score, incorporating paracenteses, cirrhosis, splenomegaly, and varices, predicts posttransplant outcomes; scores ≥ 2 associate with reduced survival [123]. Predictors of posttransplant mortality include prolonged time from Fontan failure to transplant, high MELD-XI score, renal dysfunction, and evidence of portal hypertension or varices [122, 125, 126].

No standardized guidelines exist for selecting CHLT over IHT; decisions rely on liver function, PHTN, HCC, and institutional expertise. IHT remains reasonable for preserved hepatic function, whereas CHLT is preferred in cirrhosis, decompensation, or malignancy. Sequential liver transplantation post-IHT is rarely performed due to high waitlist mortality. Multidisciplinary evaluation is essential, and standardized FALD staging and transplant criteria are urgently needed to optimize outcomes and organ allocation.

### Protein Losing Enteropathy Pathophysiology, Prevalence and Risk Factors

Protein-losing enteropathy (PLE) in Fontan patients results from excessive gastrointestinal protein loss due to multifactorial mechanisms. Central to pathophysiology is lymphatic insufficiency caused by chronically elevated central venous pressure (CVP), increased lymph formation, surgical lymphatic injury, and congenital or acquired lymphatic abnormalities [127, 128]. Persistent high venous pressure leads to lymphatic overload, dilation, abnormal lymphangiogenesis, and rupture of intestinal lymphatic vessels, allowing protein-rich lymph to enter the gut lumen [128–130].

PLE can occur even with near-normal Fontan hemodynamics, indicating central venous hypertension alone is insufficient [131]. Low serum protein reduces oncotic pressure, promoting edema and artifactually low hemodynamic measurements during cardiac catheterization [132]. Additional contributors include reduced enterocyte heparan sulfate, intestinal ischemia from low cardiac output and increased mesenteric resistance, and small intestinal inflammation with cytokine activation [133–135].

PLE affects 3–5% of Fontan patients, typically arising a median of 5 years post-surgery [129, 136]. Five-year survival has improved from ~ 50% in early reports to ~ 88% in contemporary cohorts [137, 138]. Risk factors include older age at Fontan, hypoplastic left heart syndrome, atriopulmonary Fontan, postoperative chylothorax, elevated Glenn or atrial pressures, and arrhythmias[136, 139], with early postoperative SVP > 12 mmHg predicting onset [140].

Despite universally elevated venous pressures, only some patients develop PLE, suggesting individual variability in lymphatic anatomy or function. Minor triggers such as infection or inflammation, as well as genetic or structural lymphatic differences, likely modulate susceptibility [135, 141]. 

### PLE Diagnosis

Clinicians should maintain high suspicion for PLE in Fontan patients with suggestive symptoms. Routine screening includes serum albumin, total protein, and fecal α1-antitrypsin (A1AT) every 3–4 years in children < 12 and every 1–3 years in adolescents/adults [14, 141].

Diagnosis is established by hypoalbuminemia (< 30 g/L) and increased fecal A1AT (spot > 54 mg/dL or clearance > 24 mL/24 h; > 56 mL/24 h with diarrhea) after excluding other causes of protein loss (i.e. hepatic, renal or intestinal diseases) [141]. Subclinical PLE may present with normal serum protein but elevated fecal A1AT. Work-up should assess reversible contributors via echocardiography, cardiac MRI/CT, catheterization, and rhythm monitoring [142]. Lymphatic imaging (T2-weighted MR lymphangiography) is recommended in refractory cases to identify lymphatic abnormalities [143–145].

### Management of PLE in Fontan Patients

Treatment of PLE is multifaceted, individualized, and aimed at correcting hemodynamic abnormalities, optimizing nutrition, reducing intestinal protein loss, and addressing lymphatic dysfunction. Stepwise evaluation includes echocardiography, cross-sectional imaging (including T2 or dynamic contrast MR lymphangiography), cardiac catheterization, and rhythm assessment. Correcting anatomic obstructions, arrhythmias, ventricular dysfunction, and pulmonary hypertension can improve symptoms, with pulmonary vasodilators such as sildenafil reducing Fontan pressure and mesenteric venous congestion [146].

Nutritional optimization is fundamental. A high-protein (> 2 g/kg/day), low-fat (< 25% calories) diet enriched with medium-chain triglycerides bypasses lymphatic transport [146]. Fat-soluble vitamins, iron, and electrolytes are commonly supplemented. Iron replacement may reduce intestinal inflammation and protein loss, improving clinical status [141].

Pharmacologic therapy includes diuretics (loop ± spironolactone), albumin infusions for severe hypoalbuminemia, and corticosteroids like budesonide to reduce intestinal permeability [141, 147, 148]. Heparin or low molecular weight heparin (LMWH) may reduce endothelial permeability, but benefits are inconsistent, and long-term use carries risk of osteopenia [149]. Octreotide can reduce lymphatic flow and intestinal loss with variable efficacy [150]. Midodrine has shown promise as rescue therapy in refractory cases, likely by enhancing lymphatic tone [151]. Dopamine infusion may transiently improve protein loss in severe or pre-transplant settings [152].

Refractory PLE may require lymphatic interventions, including selective embolization of abnormal hepatic or mesenteric lymphatics [128], or novel decompressive procedures such as innominate vein turn-down or thoracic duct–atrial diversion [145, 153, 154]. Surgical options—Fontan takedown, diaphragmatic plication, or systemic outflow relief—are reserved for select patients [154–156]. Catheter-based stenting or Fontan fenestration can relieve obstruction and improve preload [157]. Heart transplantation remains definitive treatment for refractory PLE, often leading to full resolution, though recovery may take up to a year. Outcomes depend on pre-transplant status and immune sensitization [158, 159].

Management must be individualized and multidisciplinary, integrating cardiology, gastroenterology, nutrition, and lymphatic expertise. Early recognition and stepwise escalation improve survival and quality of life in Fontan patients.

### PLE Outcomes

Fontan-associated PLE ranges from mild or subclinical hypoalbuminemia to progressive, refractory disease, often requiring close monitoring [141]. Treatments may induce remission, though relapse is common. Early detection and management have improved 5-year survival from ~ 50% to 88% [138, 160, 161]. Adverse outcomes correlate with elevated pressures, ventricular dysfunction, arrhythmia, and functional decline, driving hospitalizations and reduced quality of life [162].

## Conclusions

FALD and PLE are complex Fontan complications with major systemic and quality-of-life impacts. Multidisciplinary care in specialized centers has improved survival, while international registries help define mechanisms and optimize therapy. Early CVP reduction, FALD/PLE staging, lymphatic interventions, and clear transplant pathways may prevent progression and improve long-term outcomes.

## Key References

- Possner M, Gordon-Walker T, Egbe AC, et al. Hepatocellular carcinoma in the Fontan circulation: A multicenter study. JACC: Heart Failure. 2023;11(11):1534–1544.
  - This multicenter study provides critical updated data on the incidence and survival of hepatocellular carcinoma (HCC) in Fontan survivors, highlighting that malignancy can occur even in non-cirrhotic patients. It emphasizes that early-stage detection via screening is the primary driver of one-year survival rates, which currently reach approximately 79%.
- Di Maria MV, Jarasvaraparn C, Schooler GR, et al. Noninvasive laboratory-based scoring systems to estimate fibrosis stage in Fontan-associated liver disease. Pediatric Cardiology. 2024;45(2):312–320.
  - This research is essential for evaluating the efficacy of traditional markers like APRI and FIB-4, concluding that while they associate with mortality, they often lack the diagnostic accuracy to replace biopsy. The study advocates for the use of more specific markers, such as the VAST score, to better predict portal hypertension and adverse clinical events.
- Silva-Sepulveda J, Simpson KE, Canter RJ, et al. Outcomes of combined heart-liver transplantation versus isolated heart transplantation in Fontan patients: A registry analysis. Journal of Heart and Lung Transplantation. 2023;42(8):1015–1024.
  - This paper provides a significant comparison of transplantation strategies, demonstrating that five-year survival is notably higher in patients with advanced FALD who receive a combined heart-liver transplant (≈86%) compared to those receiving an isolated heart transplant (≈52%). It serves as a foundational guide for multidisciplinary teams when determining transplant eligibility and organ allocation.

## Data Availability

No datasets were generated or analysed during the current study.

## Abbreviations

FALD: Fontan-associated liver disease
PLE: Protein-losing enteropathy
HCC: Hepatocellular carcinoma
TCPC: Total cavopulmonary connection
APC: Atriopulmonary connection
CVP: Central venous pressure
FF: Fontan failure
LSEC: Liver sinusoidal endothelial cells
HSC: Hepatic stellate cell
pIVC: Partial ligation of the inferior vena cava
PHTN: Portal hypertension
VAST score: Varices, Ascites, Splenomegaly, Thrombocytopenia
BNP: Brain natriuretic peptide
TJLB: Transjugular liver biopsy
EASL: European Association for the Study of the Liver
US: Ultrasound
CT: Computed tomography
MRI: Magnetic resonance imaging
IVC: Inferior vena cava
HV: Hepatic veins
PV: Portal venous
ALT: Alanine aminotransferase
INR: International Normalized Ratio
GGT: Gamma-Glutamyl Transferase
APRI: AST-to-platelet ratio index
FIB-4: Fibrosis-4
MELD: Model for End-Stage Liver Disease
CD: Cluster of differentiation
SWE: Shear-wave elastography
MRE: Magnetic resonance elastography
LSM: Liver stiffness measurement
TE: Transient elastography
FNH: Focal nodular hyperplasia
AFP: Alpha-fetoprotein
HVPG: Hepatic venous pressure gradient
GEV: Gastroesophageal varices
CHLT: Combined heart-liver transplantation
IHT: Isolated heart transplantation
LT: Liver transplantation
A1AT: α1-Antitrypsin
MCT: Medium-chain triglyceride
LMWH: Low molecular weight heparin
